# Formative research to co-develop a Yoga-based intervention for antenatal depression in rural women in Maharashtra, India.

**DOI:** 10.12688/wellcomeopenres.25422.2

**Published:** 2026-06-03

**Authors:** Rahul Shidhaye, Kalyani Shinde, Himanshu Kulkarni, Chitra Thanage, Swapnali Palande, Shamal Talole, Unnati Thete, Pooja Nagarkar, Suryabhan Gore

**Affiliations:** 1 Professor, Department of Psychiatry, Pravara Institute of Medical Sciences (Deemed to be University), Loni, Maharashtra, India; 2 Visiting Researcher, Department of Health, Ethics, and Society, Maastricht University Care and Public Health Research Institute, Maastricht, Limburg, The Netherlands; 3 Research Assistant, Research and Develoment Cell, Clinical Trial Centre, Pravara Institute of Medical Sciences (Deemed to be University), Loni, Maharashtra, 413736, India; 4 PhD Scholar, Social and Behavioural Sciences, University of Amsterdam, Amsterdam Institute for Humanities Research, Amsterdam, North Holland, 1012 WX, The Netherlands; 5 Project Coordinator, Research and Develoment Cell, Clinical Trial Centre, Pravara Institute of Medical Sciences (Deemed to be University), Loni, Maharashtra, India

**Keywords:** Yoga, Co-Development, Theory of Change, Antenatal, Depression, Human-Centred Design

## Abstract

**Background:**

Yoga is increasingly promoted within India’s public health system as an evidence-based mind–body intervention. However, its sustained adoption and effectiveness depend on designing culturally relevant and contextually appropriate interventions. This study aimed to co-develop a Yoga-based intervention for antenatal depression in rural women and iteratively refine pathways for its effective delivery in real-world settings.

**Methods:**

We conducted a multi-phased, mixed-methods study grounded in the principles of Human-Centred Design (HCD). The study incorporated: (i) qualitative in-depth interviews with pregnant women, (ii) a series of Co-Development Workshops, (iii) an Intervention Cohort to assess feasibility, (iv) community engagement activities, (v) a Theory of Change workshop, and (vi) a stakeholder feedback session. Quantitative data were summarised descriptively, and qualitative data were audio-recorded, transcribed, translated, and thematically analysed using NVivo 9. Insights from all phases were triangulated to develop Yoga-Sanskar intervention.

**Results:**

Across all activities, participants included pregnant and postnatal women, family members, community health workers, Yoga instructors, and project staff. Participants consistently reported physical discomfort, fatigue, emotional distress, and household responsibilities as key challenges during pregnancy. Co-development workshops generated context-specific solutions, refined the Yoga sequence, and informed the design of user-friendly resource materials. The intervention cohort demonstrated feasibility and acceptability of Yoga practice. Stakeholder feedback confirmed the clarity, cultural appropriateness, and practicality of intervention materials, leading to final refinement of the Yoga-Sanskar intervention package.

**Conclusions:**

This formative research process enabled the systematic co-development and adaptation of a culturally appropriate Yoga-based intervention that aligns closely with the lived context of pregnant women in rural settings. Our approach demonstrates how human-centred, evidence-informed models can guide the design, refinement, and real-world testing of interventions to enhance their feasibility, acceptability, and implementation potential.

## Introduction

Yoga, an ancient Indian discipline grounded in indigenous knowledge systems, emphasizes a holistic integration of physical, psychological, and spiritual well-being. Originating in India over 3,000 years ago, its principles have influenced traditional and contemporary healthcare practices across Asia ( Sengupta, 2012). In recent decades, Yoga has gained renewed scientific attention, with a rapidly expanding evidence base supporting its role as a mind-body intervention for stress reduction, anxiety, depression, and improved quality of life ( Telles *et al.*, 2012). Alongside academic inquiry, national initiatives in India, particularly from the Government of India’s Ministry of AYUSH (Ayurveda, Yoga, and Naturopathy, Unani, Siddha and Homoeopathy) have prioritised integration of Yoga within the existing public health program ( Saxena *et al.*, 2024).

An increasing number of studies have examined the effects of Yoga on maternal mental health, including antenatal depression, a condition with significant consequences for both mother and child ( Punsuwun *et al.*, 2025). A culturally grounded, non-pharmacological approach may be particularly useful in India, where structural barriers, stigma, and limited mental health resources impede access to conventional psychological treatments ( Zeng *et al.*, 2025). In this context, the YOGA-D (Yoga-based Lifestyle Intervention for Antenatal Depression) program was initiated to explore the potential of a Yoga-based intervention for improving mental health among pregnant women in rural Maharashtra ( Shidhaye *et al.*, 2024). YOGA-D is a five-year, multi-phase research program comprising an initial formative research phase to co-develop the intervention, followed by a pilot randomized controlled trial to assess its feasibility and acceptability, and a subsequent pragmatic randomized controlled trial to evaluate its effectiveness and cost-effectiveness.

This paper presents the rationale, process, and key findings from the formative research phase. The primary output of this phase is the *Yoga-Sanskar* (YS) intervention (described below), which will be further evaluated for its feasibility, acceptability, and effectiveness. The protocol for the pilot randomized controlled trial has been published previously ( Shidhaye *et al*., 2024), and clinical trial registration details for the pragmatic randomized controlled trial are available online ( Clinical Trials Registry of India, 2024).

### Rational for intervention co-development

The successful implementation of an intervention depends not only on its clinical efficacy but also on the cultural fit and feasibility within women’s lived environments **.** Pregnant women in rural India experience distinct social, economic, and household constraints that may limit their capacity to engage in behavioural interventions, including Yoga ( Shidhaye *et al.*, 2023). Therefore, understanding perceptions of antenatal depression, family influence, existing coping approaches, and conceptualizations of Yoga becomes crucial for tailoring delivery methods. A formative, human-centred research approach — focused on the *user* and her *context* — offers a systematic way to explore these factors and involve participants in co-developing an intervention that reflects their felt needs and priorities ( Nijagal *et al.*, 2021).

Additionally, using Theory of Change (ToC) approach provides a framework to support the development of a clear program theory, implementation requirements, and expected outcomes ( Breuer *et al.*, 2016).

We used the Integrated Approach to Yoga Therapy (IAYT) during Pregnancy ( Satyapriya *et al.*, 2013), as an initial evidence-based intervention and collaboratively adapted its content to co-develop the *Yoga-Sanskar* (YS) intervention. IAYT module for pregnant women was developed based on classical texts, including the Patanjali Yoga Sutras and the Mandukya Karika. It adopts a holistic approach to health, addressing physical, mental, emotional, and intellectual well-being, and uses specific techniques to enhance mental equilibrium. Participants were trained in the complete Yoga sequence by certified instructors through structured sessions of two hours per day, three days per week, over one month. Refresher sessions were conducted during routine antenatal visits, and participants were encouraged to continue regular home practice using recorded materials. The IAYT intervention demonstrated significant reductions in anxiety and depressive symptoms among pregnant women ( Satyapriya *et al*., 2013).

IAYT intervention was implemented and evaluated in an urban, hospital-based setting with intensive initial training and facility-based Yoga sessions. This is markedly different from the rural context where we intend to implement the intervention. Hence, our formative research had the following objectives:

1. To understand the perceptions of antenatal depression and attitudes towards Yoga among pregnant women, families, and healthcare providers;
2. To co-develop an intervention that is feasible, acceptable, and responsive to users’ lived context;
3. To construct a Theory of Change that outlines intended pathways to outcomes;
4. To iteratively develop, test, and refine intervention materials and delivery strategies through community feedback and engagement.

## Methods

### Theoretical framework

**Figure 1.**
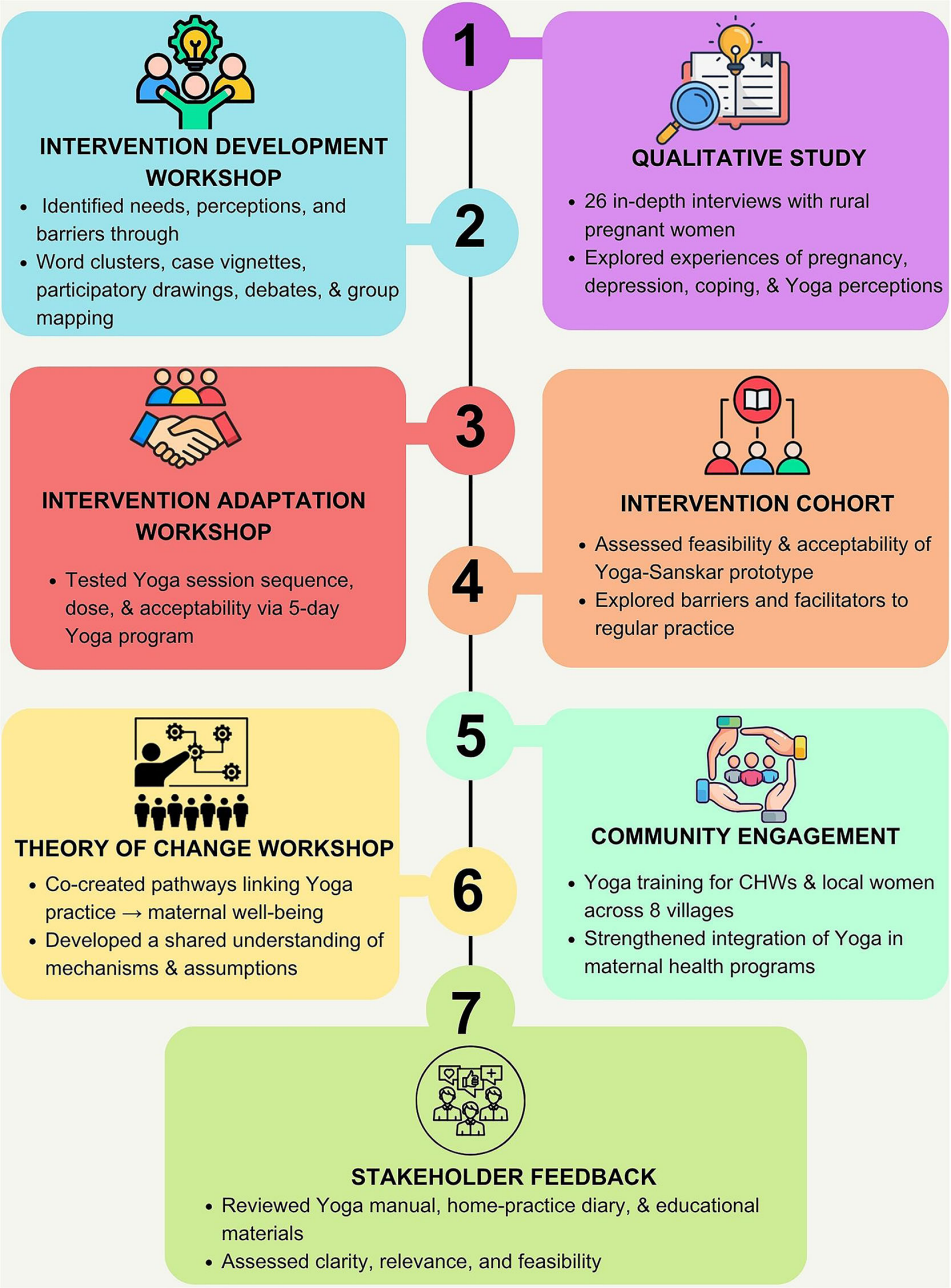
Overview of Formative research methods.

Our work was guided by the principles of Human-Centered Design (HCD) ( Kang *et al.*, 2025). By iteratively engaging pregnant women, their family members, community health workers, Yoga instructors, experts in the field of Yoga research, and a senior obstetrician, we progressed through the Human-Centered Design phases of empathizing, defining needs, ideating solutions, prototyping intervention components, and testing them in real-world settings. This process ensured that the final Yoga-based intervention ( *Yoga-Sanskar *) was grounded in the lived realities of its intended users. The overall methodology is presented in Figure 1.

### Study design

Formative research can be described as a multi-phase, mixed-methods study.

We undertook formative research from August 2022 to April 2024. It consisted of the following activities: 1) Qualitative Study (QS), 2) Co-Development Workshops (Intervention Development Workshops (IDWs) and Intervention Adaptation Workshops (IAWs), 3) Intervention Cohort (IC), 4) Community Engagement Activities, 5) Theory of Change Workshop (ToC), 6) Synthesis of Findings and Development of Intervention Materials, and 7) Stakeholder Feedback.

Qualitative study (in-depth interviews with 26 pregnant women) and the first half of the Intervention Development Workshops (described below), provided input for the first two phases of the Human-Centered Design (Empathize and Identify). Generation of ideas for improving the uptake and practice of the YS intervention occurred during the second half of the Intervention Development Workshops, Intervention Adaptation Workshops, and the ToC workshop. The YS intervention prototype was piloted for feasibility and acceptability in the IC. The data from these phases were collated, triangulated, and synthesised, leading to the development of a range of intervention delivery materials and research outputs. In the last phase, feedback from key stakeholders on intervention delivery materials was obtained, and the YS intervention was finalized.

### Qualitative study

As part of the formative phase, a qualitative study was conducted to explore rural pregnant women’s experiences of pregnancy and antenatal depression, their perceived needs and coping strategies, and perceptions, barriers related to practicing Yoga during pregnancy. A purposive sampling approach was used to recruit participants through local Anganwadi centres and primary health facilities. Anganwadi centres are community-based centres established under the Integrated Child Development Services (ICDS) programme that provide a range of essential health and nutrition services, including basic health check-ups, referrals, supplementary nutrition, immunisation, and non-formal pre-school education. Their primary beneficiaries include pregnant and lactating women, children, and adolescent girls.

Data were collected through in-depth interviews using a semi-structured interview guide. In total, 26 in-depth interviews were conducted with pregnant women diagnosed with and without depression, as assessed using the Edinburgh Postnatal Depression Scale (EPDS) questionnaire. All interviews were conducted in Marathi, audio-recorded with consent, and transcribed verbatim. Detailed methodology and findings of this study are published elsewhere ( Shidhaye *et al.*, 2025).

### Intervention Development Workshops

The primary objective of the Intervention Development Workshops was to explore the felt needs of pregnant women, along with their perceptions, attitudes, and beliefs about Yoga, as well as their overall readiness to incorporate Yoga practice into their daily routine. We conducted seven Intervention Development Workshops with a total of 124 participants, including 57 pregnant women, 21 relatives of pregnant women (such as mothers-in-law and sisters-in-law), and 56 community health workers. Further details of the participants are provided in supplementary file 1. The five key activities undertaken as part of the Intervention Development Workshops were conducted following an initial ice-breaking session and were structured as follows:

***Word cluster activity (Problem and solution mapping)***

The workshops commenced with a word cluster exercise (adapted from participatory elicitation and card-sorting methods ( Meagher *et al.*, 2024) to facilitate participants’ self-identification of problems experienced during pregnancy. Participants were presented with a set of words representing common physical, emotional, and contextual challenges, including physical symptoms (e.g., nausea, back pain), emotional states (e.g., persistent sadness, irritability, or anxiety), and contextual barriers (e.g., financial instability, household conflicts, lack of family support, agricultural work). Participants were invited to select the words that best reflected the challenges they or women in their community typically encountered.

Subsequently, a solution-oriented word cluster was presented, containing options such as resting, walking, engaging in physical activity, or consulting healthcare providers. Participants were asked to identify solutions they considered most appropriate for addressing the prioritized barriers.

***Case vignette discussion (exploration of maternal mental health perceptions)***

Following the word cluster activity, participants were presented with a case vignette depicting a rural pregnant woman exhibiting symptoms consistent with depression. This was followed by a facilitated discussion aimed at identifying the problem, exploring potential causes, and considering strategies for family and community support. The vignette served multiple purposes: it introduced the concept of depression in a culturally relevant and accessible manner, anchored abstract mental health constructs in a relatable scenario, and engaged participants in reflective dialogue.

***Visual participatory elicitation exercise (conceptualization of Yoga)***

To explore participants’ conceptualization of Yoga, we conducted a visual participatory elicitation exercise inspired by projective and concept-mapping techniques. Participants were presented with randomly selected words (e.g., “peacock,” “tree,” “parrot”, “fruit”, “school”) and the specific word “Yoga.” They were invited to write or sketch anything that came to mind in relation to each word. Facilitators encouraged participants to reflect on the meanings, emotions, and personal or cultural associations elicited by Yoga, and to compare these with the associations for the other words. Following the writing/drawing/sketching activity, participants engaged in a discussion, sharing the rationale behind their illustrations and interpretations.

***Structured participatory debate***

To explore attitudes, beliefs, and potential concerns toward Yoga practice during pregnancy, we conducted two structured participatory debates as part of the Intervention Development Workshops. The first debate focused on the perceived benefits and harms of practising Yoga during pregnancy, and the second debate addressed the question of why someone might like or dislike practising Yoga, aiming to elicit emotional and motivational drivers, perceived barriers, and the social meanings attached to Yoga practice. Each debate was moderated by a trained facilitator, with participants encouraged to adopt opposing viewpoints irrespective of their personal opinions.

***Group-Based Discussion on Barriers to Practising Yoga at Home***

The final activity comprised a group-based barrier–solution mapping exercise, designed to systematically identify obstacles to routine home-based Yoga practice and co-develop contextually appropriate strategies to address them. Participants, working in small groups, considered daily routines, household responsibilities, social norms, and environmental factors, and proposed solutions that seemed appropriate to them. This exercise functioned as a summative participatory method, integrating insights from preceding activities.

### Intervention Adaptation Workshops

The insights from Intervention Development Workshops informed the subsequent design of the Intervention Adaptation Workshops, which were conducted to assess the acceptability and adaptability of the proposed YS intervention within participants’ everyday contexts and community environments.

The Intervention Adaptation Workshops were conducted between March 2023 and August 2023 with the same participants who had attended the Intervention Development Workshops. Pregnant women were invited to participate in a five-day Yoga program, comprising daily sessions facilitated by certified Yoga instructors. The workshops were attended by 43 participants aged 18–35 years, with a mean age of 23.6 years (SD = 3.3). Further details of the participants are provided in supplementary file 1.

This phase was designed to move beyond the conceptual exploration conducted during the Intervention Development Workshops and to examine participants’ assessment of the dose, intensity, and frequency of Yoga sessions. Following the Yoga sessions, participant feedback was collected through structured questionnaires assessing multiple domains of intervention delivery, including the inclusion and sequencing of *asanas*, perceived difficulty, and overall flow. Participants also completed Yoga feedback forms to evaluate instructor performance, clarity of instruction, and satisfaction with the session delivery. Additionally, a focused group discussion (FGD) was conducted to elicit experiential reflections and collective recommendations.

### Intervention cohorts

Following the adaptation workshops, participants were invited to be part of an IC to evaluate the feasibility and practical implementation of the refined Yoga intervention. Eligible participants were pregnant women aged above 18 years, with a gestational age of 8–26 weeks, residing in the study areas, and fluent in Marathi. Women with a history of spontaneous abortion, medical advice for bed rest, or ongoing treatment for depression were excluded.

A total of 72 participants were invited to join the intervention cohort, of whom 45 agreed to participate (62.5%). A trained Yoga instructor conducted group Yoga sessions in Anganwadi centres. Each session lasted approximately 60–70 minutes, with five consecutive daily sessions in the first week followed by weekly sessions thereafter. About half of the participants (23) attended between three to five sessions, while the remaining participants attended only one to two sessions. Additionally, 20 participants attended between one to six weekly follow-up sessions. Attendance was monitored through session records maintained by the study team. To assess the acceptability and participant experience of the intervention, in-depth interviews were conducted with nine participants following completion of the follow-up Yoga sessions. These interviews were analyzed separately from the adaptation workshop data. A more detailed feasibility assessment, including process evaluation, is being conducted as part of a pilot randomized controlled trial, for which the protocol has been published ( Shidhaye *et al*., 2024) and the findings the pilot randomized controlled trial are currently under review ( Clinical Trials Registry of India, 2024) . Further details of the participants are provided in supplementary file 1.

### Community engagement activities

As part of the community engagement component, structured Yoga training sessions were conducted for various community health stakeholders across eight rural villages in the study area. The target groups included Anganwadi workers, ASHA (Accredited Social Health Activist) workers trained female community health activists under India’s National Rural Health Mission, and PHC (Primary Health Centre) health staff. Details of the participants are provided in supplementary file 1. Trained Yoga instructors conducted multiple 10-day yoga sessions using the Common Yoga Protocol, published annually by the Ministry of AYUSH. These sessions aimed to promote physical and emotional well-being, while also strengthening the role of community health workers in integrating Yoga-based practices into maternal and community health initiatives. At the end of the Yoga sessions, we conducted Focused Group Discussions (FGDs) with community health workers to understand their experience of practicing Yoga and their opinion on incorporating Yoga as part of pregnant women’s daily routine.

### Theory of Change (ToC) workshop

The ToC workshop was conducted as a participatory, structured exercise to identify the key pathways, assumptions, and contextual factors linking the YS intervention to its intended outcomes based on the feasibility and acceptability of intervention delivery. The workshop aimed to make explicit the hypothesized mechanisms of change, surface contextual enablers and barriers, and align the intervention design with stakeholder insights.

A total of 28 participants attended the ToC workshop. This group included six pregnant women, three postnatal women who had previously participated in the Intervention Development Workshops and Intervention Adaptation Workshops, three family members (spouses or relatives), four Anganwadi workers, one community facilitator involved in field-level coordination, and three Yoga instructors associated with the program. The remaining eight participants comprised project team members, including field researchers, coordinators, and the principal investigator.

The workshop was conducted in person at the Clinical Trial Centre of Pravara Institute of Medical Sciences and lasted approximately 2-3 hours. Discussions were conducted in the local language (Marathi) to facilitate active participation from all the stakeholders. Field notes were taken by trained researchers, and the ToC diagrams developed by each group were documented for analysis.

The session commenced with a brief orientation on the ToC framework by the PI who facilitated the workshop. A hypothetical example was presented to illustrate the process of back-mapping from a defined long-term outcome to its necessary preconditions. This was followed by a detailed explanation of how ToC facilitates causal reasoning in complex community-based interventions.

Subsequently, participants were divided into three groups, each guided by trained co-facilitators with prior orientation to ToC methodology. All groups were assigned a common outcome statement: *“Pregnant women practicing Yoga regularly for three months.”* Each group constructed a preliminary ToC diagram that included the following components: (i) activities and outputs required to initiate change, (ii) short- and intermediate-term outcomes, (iii) long-term intended outcomes, and (iv) underlying assumptions and barriers that could influence the causal pathway.

Each group presented its ToC map to the larger group in a plenary discussion. Feedback was solicited to identify points of convergence and contextual variation across groups. The three ToC maps were then synthesised by RS (first author) into a single consolidated version, which the co-authors subsequently reviewed for internal consistency and conceptual coherence.

ToC was primarily used to describe the programmatic aspects and contextual factors, and not to explain the biological mechanism of action. It is likely that regular Yoga practice may reduce stress through the modulation of the hypothalamic-pituitary-adrenal axis. Psychosocial mechanisms such as improved mindfulness and self-awareness (mind–body awareness), enhanced emotional regulation (self-regulation), and increased physical self-efficacy and fitness may contribute to improvements in mood and quality of life, as described by Butzer *et al*. (2016). These mechanisms are described in detail in our previously published protocol ( Shidhaye *et al*., 2024) and will be assessed as part of the randomized controlled trial.

### Synthesis of findings and development of intervention materials

We first documented the findings from co-development workshops, intervention cohorts, and community engagement activities using detailed field notes, participant outputs (e.g., participatory exercises), and facilitator reflections. The next step was synthesis of findings, and this was done using iterative team discussions. Findings from each data source were compared and contrasted to identify areas of convergence, complementarity, and divergence. For example, themes related to barriers, facilitators, and perceptions of Yoga identified in qualitative interviews were cross-checked with insights emerging from co-development workshops and intervention cohorts. This process helped us to understand context-specific nuances that informed intervention design. Triangulated findings were then mapped onto the outcomes’ pathway within the Theory of Change framework. These insights directly informed the development of intervention delivery strategies, session content, and supporting materials (e.g., Yoga-Sanskar chart, *Yoga-Dainandini,* and digital resources). This integrative approach ensured that the final intervention was grounded in both empirical evidence and participant- driven insights. The details are in the results section below.

### Stakeholder feedback

Following the development of the intervention materials, a workshop with key stakeholders was conducted to evaluate the clarity, relevance, and acceptability of the proposed Yoga-based intervention materials. A total 11 participants attended the Stakeholder Workshop, including two community facilitators, two postnatal women, two antenatal women, three Anganwadi workers, and two Yoga instructors.

During the workshop, the research team presented the finalised intervention materials, which included a Yoga sequence, a Yoga session manual, a home practice diary, Yoga charts, and health education materials. Each material was demonstrated and discussed in detail to elicit participants’ perceptions regarding content comprehensibility, cultural appropriateness, visual appeal, and feasibility of implementation within community settings. Quantitative feedback was collected using a structured feedback form developed by the research team.

### Data management and analysis

Quantitative data were collected through various activities conducted during the Intervention Development Workshops, Intervention Adaptation Workshops, Intervention cohorts, Community Engagement Activities, and stakeholder feedback. These activities generated data on pregnant women’s needs, coping strategies, and perceptions of Yoga during pregnancy, as well as structured feedback on session sequence, intensity, frequency, and instructor performance. Additional inputs were obtained through satisfaction forms, validation of intervention components from the Intervention cohorts, and stakeholder feedback on intervention materials. Quantitative responses were systematically compiled, entered, and analyzed descriptively to summarize and identify areas for refinement in intervention design and delivery.

Qualitative data from In-depth Interviews and Focused Group Discussions were audio-recorded, transcribed verbatim, and translated from Marathi to English. We employed thematic content analysis to examine and synthesize the data ( Anderson, 1997; Braun & Clarke, 2012). Members of the research team (RS, ST, SP, KS, and PN) repeatedly reviewed the transcripts in both English and Marathi to achieve familiarity with the data and met regularly to discuss emerging insights. An initial coding framework was developed after SP and ST independently coded two transcripts. This draft codebook was subsequently reviewed and refined in consultation with RS. Coding responsibilities were then divided: SP independently coded approximately half of the transcripts, which were later reviewed by ST, while ST coded the remaining transcripts, followed by review from SP. Additional codes were generated inductively as new patterns emerged from the data. Any discrepancies in coding were discussed and resolved collaboratively among RS, SP, and ST. The broader research team (RS, KS, SP, ST, PN, and SG) participated in reviewing the coded data and identifying key themes. NVivo 9 (QSR International) was used to organize, manage, and analyze the data throughout the process.

### Reflexivity and positionality

The data were collected, transcribed, translated, coded, and analyzed by a team of researchers familiar with the local cultural context. CT conducted all IDIs and FGDs. She holds a master’s degree in medical social work and clinical psychology and is currently pursuing a doctoral degree. At the time of data collection, she also served as the intervention coordinator, overseeing the intervention adaptation process. CT is a native Marathi speaker and shares the sociocultural background of the participants, which facilitated rapport-building and contextual understanding. She had prior experience in conducting structured interviews and received additional training from RS in qualitative data collection methods.

Other members of the research team (KS, SP, ST, PN, and SG) are also native Marathi speakers with a strong understanding of the local context and were involved in various stages of qualitative data analysis. RS has previous experience in leading qualitative research studies.

At the beginning of each interaction, CT introduced herself and explained the study objectives to participants. While her dual role as a researcher and intervention coordinator may have influenced participants’ responses and data interpretation, efforts were made throughout the research process to maintain reflexivity through team-based discussions, iterative analysis, and critical review of emerging findings.

### Ethics and consent

Ethical approval for this study was obtained from the Institutional Ethics Committee of Pravara Institute of Medical Sciences, Loni, Maharashtra, India (Approval No.: PIMS/DR/RMC/477). The study involved formative research with human participants and was conducted in accordance with the ethical standards of the Institutional Ethics Committee and the principles of the Declaration of Helsinki (1964) and its later amendments.

All eligible participants received a participant information sheet that provided detailed information about the study objectives, procedures, and the contact details of the Principal Investigator and the Member Secretary of the Institutional Ethics Committee. Participants were given adequate time to review the information and to ask questions prior to participation.

Written informed consent was obtained from all participants who agreed to take part in the study. For participants who were unable to read or write Marathi but could communicate verbally in Marathi, the participant information sheet was read aloud. Verbal informed consent was obtained and audio-recorded, and a thumb impression was taken in lieu of a signature. The consent process for such participants was witnessed by an impartial third party (e.g., a family member or healthcare worker), who also signed the consent form.

### Public and patient involvement

Patient/participant involvement was central to the work described in this paper. The primary aim was to co-develop the Yoga-Sanskar intervention through close engagement with pregnant women, their family members, community health workers, Yoga instructors, and experts in Yoga research. Participants’ perspectives directly informed the Theory of Change workshop, the identification of strategies to address barriers to regular Yoga practice, and the design of intervention materials. These participatory processes and their contributions are described in detail in the results section below.

## Results

### Findings of intervention development workshops

Low backache, fatigue, headache, and irritability were reported as the most commonly occurring problems during pregnancy (about 50%). A third to a quarter of the participants cited sleep disturbances, nausea and vomiting, sadness and/or anxiety, extreme anger, crying episodes, boredom, and not feeling like doing anything as the commonly encountered problems.

Few participants (about 10%) reported household chores, interpersonal conflicts within family and pressure to deliver a male child as their concerns.

Most of the pregnant women reported taking rest (‘ *aaram karne*’)/relaxing as the best way to cope up with their problems. About half of the participants said that they go to a temple, go for a walk, watch content on their mobile phone, read, or consult a doctor to feel better.

Discussing their problems with their spouse/mother, having a chat with neighbours or friends, avoiding verbal conflicts within the family, or taking a nap were suggested as strategies to deal with stress by about a third of the participants. About 10% of the participants thought that Yoga was one of the solutions to deal with stress.

Yoga was commonly conceptualized as a form of physical activity that involves movement of upper and lower limbs, activities related to breathing such as deep inspiration, deep expiration, and holding breath, and recitation of ‘ *Omkar*’. *Suryanamaskar*, *Padmasan*, *Pawanmuktasan*, *Utkatasan*, *Konasan*, *Parvatasan* were names of some of the asanas that participants were aware of. They also equated Yoga with the activities *‘Shri Ramdev Baba’* performs. They had watched Yoga on TV. In one workshop, participants mentioned that Yoga was the union of the individual soul with the universal soul. Some participants mentioned that even going for a walk or jogging was a form of Yoga.

More than half of the participants mentioned that practising Yoga helps in reducing psychological stress, improving mental health, as well as overall health (‘ *aarogya*’).

A third of the participants felt that regular practice of Yoga leads to happiness, positivity, and a general sense of well-being. Additionally, some participants stated that Yoga reduces the probability of preterm labor, increases the probability of normal delivery, reduces problems during delivery, and improves health of the fetus.

The most commonly reported barrier to Yoga practice was a lack of knowledge about how to practice Yoga (about 30%), followed by household responsibilities (20%). Additionally, less than 15% participants reported barriers such as fatigue and minor ailments, boredom, lack of time, and space constraints. A smaller proportion (less than 10%) of participants cited childcare responsibilities and family disapproval or lack of permission as barriers. Less frequently mentioned barriers included shyness, clothing-related discomfort ( *saree*), beliefs that Yoga may harm the baby, and low motivation.

Most common suggested facilitator by participants was enhancing knowledge about the benefits of Yoga (15%). Less than 10% participants frequently mentioned strategies included planning the day ahead, seeking permission from family members, and developing a personal interest in Yoga. Additionally, same proportion of participants emphasized allocating dedicated time for Yoga practice and using the internet for guidance or motivation.

A few (less than 10%) participants perceived Yoga as potentially harmful to health, particularly during pregnancy. The most commonly cited concern was a lack of knowledge about correct Yoga practices. Several participants expressed fears that Yoga might harm the baby, including risks of low birth weight, abortion, or preterm labor. About 5% mentioned that improper or excessive Yoga practice could cause physical distress or injury or be detrimental to both the mother and baby. Some women also associated Yoga with excessive exertion and reported that it could interfere with household responsibilities.

The feedback from participants indicated a highly positive experience with the Intervention Development Workshops. Participants described the workshop as *inspiring*, *pleasant*, and *motivating*, reflecting strong engagement and satisfaction with its content, facilitation, and organization. Overall, the workshops were well-received, enhanced participants’ understanding of Yoga, and fostered motivation and family support for Yoga practice during pregnancy.

### Findings of intervention adaptation workshops

The majority of participants consistently recommended including all components, especially the Opening and Closing Prayer, and *asanas* such as *Tadasana, Ardha-kati Chakrasana, Trikonasana, Vajrasana, Vakrasana, Siddhasana, and Baddha-konasana.* They also recommended Breathing Practices, including Deep Breathing, *Nadi Shuddhi, Sheetali*, and *Bhramari*, as well as Relaxation postures like *Shavasana.* Across all components, most participants responded with “Definitely Included”. Participants did not suggest any changes to the Yoga sequence taught by Yoga instructors, as they didn’t face any difficulty practising Yoga. Notably, four participants expressed their view to include more *asanas* in the sequence.

Overall, participants found Intervention Adaptation Workshops to be very good. All aspects of the Yoga session, from content to delivery, were highly appreciated by the participants. The only “Can’t say” in the entire feedback indicates a clear, memorable experience for almost all participants. Participants also described the workshop as *healthy, inspirational, attractive, pleasant, enjoyable, and helpful.* Only two participants felt it was *boring* and *overwhelming.*

### Findings from the qualitative data

The IDIs provided rich insights into participants’ experiences with the Yoga sessions. The findings are summarized in Table 1 across four major themes: (a) feedback on the sessions; (b) benefits of Yoga; (c) barriers to participation; and (d) readiness for continued practice.

**Table 1.** Summary of the findings from the In-Depth Interviews and Focus Group Discussions.

Theme	Sub-Theme	Indicative Quotes
**Feedback on the Sessions**	Trainer support and instruction quality	*“First of all, I sincerely thank Pravara Institute for making this Yoga training available to us. The trainers teach us very well. Because no one has taught us with such sincerity before, not even after paying. No one has ever taught us like this with such heart. The main thing is that while teaching, you don’t just teach asanas; you also teach how to warm up first, and Sir himself says that you should do it for yourself. This is an important point we’ve realized.”* (CHW_FGD_Shrirampur) *“Their method of teaching was great. If we do not understand any asana then they tell us again. They tell about the benefits of Yoga and diet. They explained how to do Yoga or How to take care while doing yoga practice.”* (22y, Graduate, PG, D+) *“She explained everything beautifully. She not only gave us information but also helped us understand how to apply Yoga in our daily lives, the benefits it brings.”* (24y, Graduate, PG, D–)
	Group participation and peer support	“ *A woman who never leaves home can come out of the house because of this project and Yoga is great for our body, everyone should do Yoga.”* (C_E_IDI_08_13_12_2023) *“If we do Yoga in the group it feels nice but if we do it alone at home then it becomes boring.”* (22y, Graduate, PG, D+) *“There are one or two women who say that we felt good and our delivery was normal, even though the baby was overweight, they had a normal delivery. Otherwise, how could it be possible to do normal delivery and not cesarean section.”* (C_E_IDI_03_24_11_2023)
	Encouraging participation and awareness	*“As I mentioned earlier, today’s generation is getting too involved with mobile phones nowadays. However, mobiles do have some advantages too. can provide guidance anytime, and we can watch videos on YouTube to attend various sessions. Through these, we can learn Yoga and even connect with each other via online sessions.”* (24y, Graduate, PG, D–) *“First of all we should go to their house, visit them, and encourage them, or any senior person in the house like their mother-in-law or Mister, to make them understand the importance of Yoga and Convince and encourage them to send pregnant women to do Yoga.”* (C_E_IDI_08_13_12_2023) *“We have to awaken them to remove their fear, then that fear in their mind goes away from their mind, they get ready by themselves.”* (C_E_IDI_05_12_12_2023_Loni) *“Pregnant women who have done Yoga can tell their experience to another woman then other pregnant women can realize the benefits of Yoga practice.”* (C_E_IDI_07_13_12_2023_Kolhar)
**Benefits**	Physical Benefits	*“I am 58 years old. Actually, I am very happy to be able to do this exercise at my age because at this age, people usually say that old age is coming. I have sugar and high blood pressure, and at this age, I used to feel tired doing anything. I used to feel like resting all the time, but now it’s not like that. Even when I go home, I feel full of energy. My body feels lighter, and I feel like I’ve become much younger. My stomach, which used to be big, now seems to have shrunk, and I feel energetic and happy.* *Honestly, I feel very energized now. I used to lie in bed and not help my daughter-in-law with anything, but now I wake up early, light the stove, Sweeps, heats the water for the bath, and help with everything. I feel fresh and good. Earlier, I couldn’t sleep well. If I woke up in the night, I would stay awake until 3 AM. But now, I sleep well, and my meals have improved too. I used to eat only half a roti, but now I eat a full roti and feel much better. At my age, people usually talk about old age, but after doing Yoga, I feel great, and it feels so nice. Plus, I don’t feel the financial burden, and especially, I can wear salwar-kurti now.”* (CHW, FGD_Shrirampur) *“when I participated in this, you must have seen my neck, that is, I was coming there with the belt on, and since then I have not held the belt for two years, and I have not seen where it fell. Totally covered by that Yoga and the Yoga I do.”* (C_E_IDI_04_12_12_2023) “ *Almost 70–80 percent has made a difference to me too with Yoga and saved my hospital expenses.*” (C_E_IDI_04_12_12_2023) “ *I think Yoga is as important as eating and drinking in life. It helps improve our body and lifestyle, especially in today's fast-paced world. People eat and drink whatever they like without caring about their health. Yoga can bring balance. Even if you're busy, just a little Yoga in the morning can make you feel fresh and happy all day.*” (26y, Graduate, Multigraavida (MG), D+)
	Health-life balance	*“During pregnancy, women often feel tired and lazy when it comes to doing household work. But with Yoga, they won’t feel that way. The increased energy and enthusiasm will positively affect the baby too. If the mother remains happy, it will have a good impact on the child. It will benefit both, even the baby’s intelligence quotient (IQ) is believed to improve.”* (CHW_FGD_Shrirampur) *“We have a woman who was childless for almost nine years, she did Yoga and immediately she became pregnant.”* (C_E_IDI_03_24_11_2023)
	Psychological benefits	*“Our mental stress was relieved and also, we got happiness.”* (C_E_IDI) *“Mental health becomes good due to exercise and does not feel irritable. Mind stays happy and the immune system improves. Also, their diet improves, they have peaceful sleep. Doing Yoga during pregnancy can be very beneficial for delivery.”* (C_E_IDI) *“During pregnancy, women face numerous challenges: mood swings, irritation, lack of appetite, sudden bouts of sadness, many of which they may not be able to share even with family members. However, considering today’s generation and the current situation of women, they are often unable to express their experiences openly. This highlights the great need for Yoga practice, not just for physical health but also for mental and emotional well- being.”* (cohort IDI Shrirampur) *“Yoga practice helps a lot for better delivery and the garbh sanskar for foetus and immunity remains good.”* (C_E_IDI_06_13_12_2023)
**Barriers to Participation**	Misconceptions about Yoga	*“Difficulties may arise like time planning at home, some of them have family problems and some misunderstandings about Yoga practice like, is it necessary to do Yoga? Is Yoga done by pregnant mothers? Family thinks that a pregnant mother works during the day from morning to evening. They think that her daily movements are her Yoga so no need to do Yoga.”* (CHW, FGD) *“I think they were having fear that if we do Yoga, it may harm their baby inside, if we raise our hands and feet, then they have a fear that something can happen.”* (CHW_FGD) *“There’s a belief that exercising might cause miscarriage or harm the baby. Some think there may be health issues that will affect the baby, so they’re hesitant to do Yoga.”* (Yoga_D_FGD_Shrirampur) *“The women are doing housework, i.e., making tiffins for their husband, washing and cleaning utensils etc.”* (C_E_IDI_03_24_11_2023) *“If it’s a joint family, it becomes even more uncomfortable for them.”* (Yoga_D_FGD_Shrirampur)
	Lack of time and space	*“We work in slum areas where the mindset is not supportive. Families don’t allow their daughters-in-law to go out.”* (Yoga_D_FGD_Shrirampur) *“There is no space at my home.”* (24y, Grade 7, PG, D+)
	Transportation	*“She needed someone to drop & pick her up, it was a long distance and the source to come was also a problem.”* (C_E_IDI_01_24_11_2023)
	Socio-cultural background	*“Muslim area. Muslim women don’t come outside.”* (Yoga_D_FGD_Shrirampur) *“The area I serve is completely uneducated. It's the mangwada, rajwada side, and those people won’t really agree. No matter how much we try, it’s very difficult.”* (Yoga_D_FGD_Shrirampur) *“When I realized that Yoga is for me and beneficial, I made time for it.”* (24y, Graduate, PG, D-)
**Readiness to do Yoga**	Family and social support	*“I practice with my husband and mother-in-law. When we gain knowledge and share it with others, it grows multifold.”* (24y, Graduate, PG, D-) *“My family supports me a lot. They tell me to finish my work and focus on Yoga. Sometimes they even ask how to do certain practices, and I guide them. My mother-in-law also joins me for poses that help with leg or back pain.”* (26y, Graduate, MG, D+) *“Dnyaneshwar Maharaj was also a yogi. We are inspired by him.”* (Yoga_D_FGD_Shrirampur) *“I was inspired by the Yoga teacher. I got interested in doing Yoga because of Yoga teacher. I was feeling lazy at home, but when she was doing it, I wanted to do it, so now I am continuing.”* (22y, Graduate, PG, D-) *“After coming for Yoga, I am feeling very good. In fact, when it’s time for Yoga, I feel excited. I don’t feel like missing it at all, No laziness, no boredom, nothing like that.”* (Yoga_D_FGD_Shrirampur) *“Almost 70–80 percent has made a difference to me too with Yoga and saved my hospital expenses.”* (C_E_IDI_04_12_12_2023)

Community Health Workers expressed strong appreciation for the trainers, noting their supportive approach and the structured inclusion of warm-up exercises- an element many found especially beneficial. Pregnant women similarly valued the sessions, describing them as holistic and practical, extending beyond Yoga postures to include advice on exercise, diet, and integrating Yoga into everyday routines. Participation in the Yoga sessions also provided women with a sense of agency and autonomy, enabling them to step outside their homes and engage in shared activities with peers. The group setting fostered solidarity, motivation, and mutual support. Some Community Health Workers reported that women specifically attributed their normal deliveries to the benefits they experienced from practicing Yoga during pregnancy.

Community Health Workers reported several physical benefits of Yoga, including improved sleep, reduced fatigue, and increased energy levels. One Community Health Workers noted that improvements in physical health reduced her need for medical visits, thereby lowering household expenses—highlighting both health and economic advantages of Yoga practice.

Beyond physical benefits, Community Health Workers emphasized positive effects on mood, including reduced irritability, enhanced emotional well-being, and a sense of mental “freshness.” They perceived these improvements as particularly valuable during pregnancy for both maternal and fetal health.

However, several misconceptions and contextual barriers limited participation. Families often questioned the need for Yoga, assuming women’s household work already constituted sufficient physical activity. Some expressed concerns about potential harm to the fetus. Structural barriers such as limited space in joint families, domestic responsibilities, festive obligations, and lack of dedicated time further constrained practice. Transportation challenges—especially reliance on family members for travel—also discouraged attendance. In some communities, including Muslim households, restricted mobility without family support limited participation.

To overcome participation barriers, participants recommended practical adjustments to session timing—preferably after 12 noon, once household responsibilities are completed—allowing women to consistently dedicate an hour to practice. They also emphasized the importance of holding sessions in familiar and easily accessible locations such as *Anganwadis* (local community health centers) or schools to improve comfort and family acceptance.

Participants suggested leveraging technology and digital media (e.g., YouTube videos demonstrating benefits of Yoga) alongside door-to-door outreach to raise awareness and reassure families. Peer influence was seen as equally important: women believed that positive experiences shared within their cohort would motivate others and highlight the value of group-based sessions.

Readiness to engage in Yoga increased when women perceived personal benefits and felt a sense of agency over their own well-being. A pregnant participant expressed that Yoga could seamlessly become part of daily life, “like eating or drinking”.

While family resistance was frequently cited—due to cultural norms, misconceptions, or household duties—there were encouraging examples of supportive involvement from husbands and mothers-in-law, which strengthened motivation.

Other enablers included guidance and inspiration from Yoga trainers, as well as spiritual associations. Overall, these insights highlight that participation is shaped by a dynamic interplay of barriers and facilitators—often rooted in the same social environment—underscoring the need for community-aligned strategies to support sustained Yoga practice.

### Synthesis of findings and development of intervention materials

The *Yoga-Sanskar* sequence was finalized through an iterative process informed by participant feedback during the Intervention Adaptation Workshops and expert review from a Yoga research specialist. Minor modifications were made to tailor the sequence for women in their second and third trimesters. To support home-based practice, two YouTube playlists were developed—one for each trimester—and a colour A3-size *Yoga-Sanskar* chart was printed, featuring images of all Yoga activities and corresponding QR codes linking to the related videos. These visuals and QR codes were also incorporated into the *Yoga-Dainandini* (daily home practice log).

A short video was created to support participant orientation by:

a) describing the role of Yoga in promoting mental health during pregnancy, addressing common misconceptions and safety concerns, and explaining the study objectives and randomization procedures.

Insights from the Intervention Adaptation Workshops led to the development of the Common Symptoms in Pregnancy (CSIP) scale to identify frequently experienced pregnancy-related symptoms. However, during piloting, the Common Symptoms in Pregnancy tool was found to be operationally challenging. Therefore, key symptom-monitoring elements were embedded within the *Yoga-Dainandini* to facilitate routine self-reflection and tracking. Additionally, a question was added to the Attitude Towards Yoga Scale (ATYS) to assess perceived changes in these symptoms over time. Yoga instructors were trained to introduce the symptom-reflection component early in the intervention, helping them build rapport, discuss individual concerns, and motivate continued engagement.

The Intervention Development Workshops also underscored a need to assess women’s understanding of Yoga during pregnancy, their likes and dislikes, and perceived barriers to regular practice. This led to the development of the Attitude Towards Yoga during Pregnancy Scale (ATYS), which aims to capture these psychosocial aspects and assess the effect of the attitude on regular practice of Yoga.

### Theory of change

**Figure 2.**
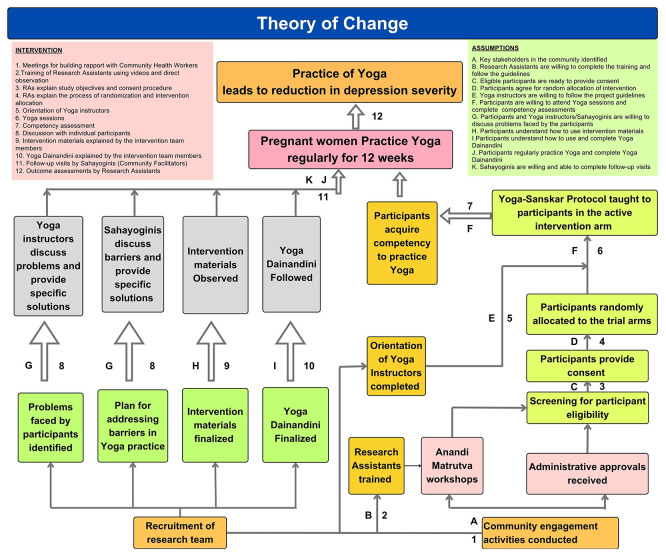
Theory of Change Map.

The final ToC consisted of an outcomes map, a set of intervention components linked to these outcomes, and the underlying assumptions along the causal pathway ( Figure 2). The ToC specified that the long-term outcome—improvement in maternal mental health—will be achieved if pregnant women engage in regular Yoga practice for at least twelve weeks. To support this behavior, women must develop sufficient competency and confidence in Yoga, engage with the intervention materials, and routinely use the *Yoga Dainandini* home-practice guide. Additionally, Yoga practice must be perceived as relevant to their felt needs/problems they routinely experience, and practical barriers to routine home-based practice must be addressed.

The key assumptions embedded in this pathway include:

a) participants’ willingness to enroll and remain engaged in the intervention;
b) consistent attendance at Yoga sessions to acquire necessary skills; and
c) sustained motivation to continue daily home practice using the *Yoga Dainandini.*

### Stakeholder feedback

Feedback from participants indicated high acceptability and usefulness of the intervention materials. Most items received median scores of 9–10, reflecting positive views on the poster, flipchart, and *Yoga Dainandini’s* clarity, design, and relevance. The highest-rated aspects included the structure of the *Yoga Dainandini*, the perceived usefulness of mental health screening for pregnant women, and the relevance of questions addressing irritability, tension, energy, and sleep. Stakeholders especially appreciated the inclusion of mental health messages and digital practice aids (QR codes, YouTube links). A few raised practical concerns about providing Yoga mats and bags. Overall, the findings suggest that the materials were well-received and appropriate for community implementation.

### Summary of adaptations made to the original intervention

Based on feedback from both experts and participants during the formative research phase, several adaptations were made to the original Integrated Approach of Yoga Therapy during Pregnancy (IAYT-P) to enhance feasibility, acceptability, and adherence in the local context. The original Yoga module was simplified to improve safety and suitability for pregnant women in community settings. Specific practices were removed, including *Gulpha vistara svasanam* (ankle stretch breathing), *Trikonasana* (triangle pose), *Ardha pavanamuktasana* (folded leg lumbar stretch), and *pranayama* techniques such as *Sheetali.* In contrast, gentle and supportive practices such as *Griva Sanchalan* (neck movements) and *Supta-baddha Konasana* were included to enhance comfort and appropriateness during pregnancy.

Findings from the intervention adaptation workshops further highlighted the need to incorporate behaviour change strategies alongside the Yoga content, leading to modifications in the structure and delivery of the intervention. The programme was reorganised into two phases: a LEARN phase and a PRACTICE phase, to support gradual skill acquisition and sustained engagement. The LEARN phase consisted of directly supervised group sessions led by trained Yoga instructors, beginning with an initial orientation session focused on building commitment, explaining the benefits and safety considerations of Yoga during pregnancy, and demonstrating the full Yoga sequence. This was followed by three additional supervised sessions conducted on consecutive days, during which participants were taught individual components of the sequence, including *sukshma-vyayam *, selected asanas, *pranayama*, and relaxation techniques, with emphasis on correct technique, breath awareness, and safety. Participants also received individualised feedback and modifications based on their comfort and ability. The PRACTICE phase began alongside the LEARN phase and extended over three months, during which participants were encouraged to progressively transition to independent home- based practice. A competency assessment was conducted within three to four weeks after the first session to ensure safe and correct practice. Weekly group sessions were conducted during this phase to reinforce learning, address challenges, and maintain motivation. The duration and structure of sessions were standardised, with supervised sessions lasting approximately 60–75 minutes. To further address barriers to adherence identified during formative research, several supportive components were integrated as described above. These included a Yoga diary ( *Yoga Dainandini*) with structured home practice guidance and self-monitoring, QR code–linked video demonstrations, a Yoga manual, and educational leaflets on physical activity, sleep, and diet during pregnancy. In addition, a trained community-based facilitator (Sahayogini) provided ongoing motivation and support through regular interactions. Together, these adaptations ensured that the intervention was contextually appropriate, participant-centred, and feasible for implementation in real-world settings.

## Discussion

In this paper, we describe the formative research process that guided the co-development of *Yoga-Sanskar *, a Yoga-based intervention for antenatal depression among rural women in Maharashtra, India. Grounded in the principles of Human-Centered Design (HCD), the study adapted the core Yoga sequence from the Integrated Approach to Yoga Therapy during Pregnancy ( Satyapriya *et al.*, 2013) through active engagement with key stakeholders. The formative phase of YOGA-D was designed to understand the ‘ *user in her context’,* the pregnant woman situated within her family, community, and healthcare environment, so that the intervention could be responsive to her lived realities. This approach underscores the value of formative research grounded in Human-Centered Design, demonstrating how centering the user enables the creation of culturally, socially, and logistically relevant interventions that promote maternal mental health in low-resource settings. Through a series of participatory workshops, we explored women’s attitudes toward Yoga during pregnancy, gathered feedback on intervention delivery, and collaboratively developed a ToC that maps the change pathway. The formative research phase led to the co-development of the *Yoga-Sanskar* intervention. We plan to evaluate the feasibility, acceptability, and effectiveness of the intervention using a pilot randomized controlled trial ( Shidhaye *et al*., 2024) and the pragmatic randomized controlled trial design ( Clinical Trials Registry of India, 2024) . The following sections discuss key learnings, strengths, limitations, and implications for future research and implementation.

First, the emphasis was on understanding our *user* (a rural pregnant woman) in her *context*, to ensure that the *Yoga-Sanskar* intervention addressed the pregnant women’s felt needs and the factors that affected their mental health and help-seeking behaviour. This perspective informed not only *what* was delivered/content (i.e., core Yoga sequence) but also *how* it was delivered (format, medium, and setting). Our findings suggest that pregnant women have a fairly good understanding of the benefits of Yoga practice during pregnancy. At the same time, they had concerns related to safety. The local socio-cultural context shaped their attitudes. The significance of context has been well-articulated in implementation science frameworks such as the PARIHS (Promoting Action on Research Implementation in Health Services) and CIFR (Consolidated Framework for Implementation Research) models, both of which highlight context as a critical determinant of successful intervention implementation ( Rycroft-malone *et al.*, 2013). Our previous qualitative study provided valuable insights into lived experiences and felt needs of pregnant women, but offered limited understanding of the broader contextual and cultural factors that would influence engagement with Yoga practice ( Shidhaye *et al.*, 2025). The Intervention Development Workshops that we conducted enabled a deeper exploration of the socioeconomic and daily-life context of pregnant women—for example, living in joint families and frequent household guests, which limited privacy and opportunities to practice Yoga at home.

Second, we actively involved pregnant women in the intervention adaptation process by seeking their structured feedback on the core Yoga sequence, the duration, frequency, timing, and the setting for the delivery of Yoga sessions. The exercise provided an applied framework to assess the practical feasibility, comfort level, and perceived appropriateness of the Yoga sessions within the local context. The activity was not intended to evaluate effectiveness but rather to identify factors influencing engagement, comfort, and perceived suitability, thereby informing refinements before integration into the larger randomized controlled trial. Unlike routine pilot or feasibility trials that often focus on procedural aspects such as recruitment, retention, fidelity, and outcome measure acceptability, our approach directly informed the final iteration of the intervention package, ensuring that it was contextually grounded, and operationally feasible for field implementation.

Third, the development of a program theory using the ToC approach enabled the synthesis of key insights from earlier workshops, the identification of critical intermediate outcomes, and the specification of intervention materials required to achieve them. The ToC framework is widely applied in the design and evaluation of public health interventions ( Breuer *et al.*, 2016) as it encourages stakeholders to explicitly articulate the assumptions underlying their program logic and to consider how contextual factors may influence implementation and outcomes.

Several limitations of this study should be acknowledged. Participant recruitment was facilitated through community health workers (CHWs), resulting in purposive sampling. Pregnant women with severe depression or medical conditions requiring bed rest were not included, and their perspectives were therefore not represented. Obstetricians and primary healthcare staff could not be involved in the workshops, limiting the diversity of stakeholder inputs. Group discussions may have been influenced by more vocal participants or by social desirability bias, although facilitators made deliberate efforts to balance participation and encourage open expression of views.

The findings are also shaped by the positionality of the research team, who were involved in data collection, analysis, and synthesis across multiple stages. Given that all participants were from rural settings with similar sociocultural backgrounds, the findings are transferable primarily to populations with comparable contexts rather than to urban pregnant women. Finally, as participants were not followed beyond three months, the study could not assess long-term adoption or sustainability of Yoga practice during and after pregnancy.

Despite these limitations, this study is unique in several ways. While co-development, Human-Centered Design (HCD), and Theory of Change (ToC) approaches are increasingly used in public health research, their application within Yoga-based interventions remains relatively underexplored. This study is one of the very few to apply these approaches in this context. Although there is growing evidence for Yoga-based interventions, there remains a gap in user-centered models of intervention. Central to effective intervention design is the principle that the user is at the heart of the process, with interventions crafted for her, alongside her. Together, these processes — user understanding, contextual inquiry, iterative feedback, and theory-building — that places the “user in her context” at the core of every stage with scientific methodology aligns with every phase of human centred design from empathy-building, define — clarifying the felt need based on contextual realities to ideate–prototype–test cycle, ensuring co-creation and iterative improvement result in evidence-informed, contextually, and User-centered intervention and materials that are outputs of this study. This highlights that the materials, session content, and delivery strategies are thus not abstractly designed but are co-developed, tested, and refined with those who will ultimately use them. Unlike other studies in the field of perinatal mental health, this study moves beyond only In-depth Interviews and Focused Group Discussions and provides a detailed description of the co-development process.

The study’s multi-method, iterative design integrated qualitative inquiry, co-development workshops, Intervention Cohort, and ToC mapping, allowing a deep understanding of user needs and system realities. The inclusion of diverse stakeholders—pregnant women, community health workers, Yoga instructors, and mental health researchers—enhanced ecological validity and acceptability. The research team’s reflexive engagement throughout data collection and synthesis further strengthened interpretive rigor. Importantly, the process led to the development of tangible, user-friendly materials, including the *Yoga-Sanskar* chart, *Yoga-Dainandini *, and short videos, enhancing the potential for translation and scalability.

This study has two key implications for the practice and research of Yoga-based interventions. First, the Government of India’s initiative to integrate Yoga into Ayushman Arogya Mandirs under the Ayushman Bharat program provides a unique opportunity to translate these findings into practice. The insights from this formative research can inform strategies to address barriers to participation, enhance community engagement, and optimize the delivery of Yoga sessions for pregnant women. Second, this study presents a comprehensive, co-development approach that integrates traditional Indian knowledge systems with contemporary participatory and theory-driven research frameworks. This model demonstrates how culturally rooted and evidence-informed interventions can be systematically developed, refined, and adapted for diverse public health and mental health contexts.

## Data availability

### Underlying data

The data of this study are openly available in Figshare.

Figshare: Formative research qualitative data available here: https://doi.org/10.6084/m9.figshare.30919667.v1.

This study contains the following underlying data:

• English translations of all In-Depth Interviews (IDIs) and Focus Group Discussions (FGDs) conducted as part of the formative research activities.

**Citation**

Shidhaye, Rahul (2025). Qualitative Data_Formative research to co-develop a Yoga-based intervention for antenatal depression in rural women in Maharashtra, India. figshare. Dataset. https://doi.org/10.6084/m9.figshare.30919667.v1

### Extended data

Figshare Repository: WOR_Formative_Research_Supplementary_File_1 https://doi.org/10.6084/m9.figshare.30665477.v2

Figshare Repository: WOR_Formative_Research_Supplementary_File_2

Figshare Repository: WOR_Formative_Research_Supplementary_File_3

Supplementary files are available here: https://doi.org/10.6084/m9.figshare.30665477.v2

This study contains the following extended data:

• Supplementary File 1: Workshop activities used during the formative research and detailed participant characteristics collected as part of the formative research.
• Supplementary File 2: Yoga-Sanskar intervention protocol
• Supplementary File 3: Intervention materials developed from formative research findings

Data are available under the terms of the Creative Commons Zero “No rights reserved” data waiver (CC0 1.0 Public domain dedication).

### Declaration of the use of AI

During the preparation of this work, the authors used the Chat Generative Pre-Trained Transformer (ChatGPT; OpenAI, San Francisco, CA, USA), version 5.1, to improve the language, flow, grammar, and overall readability of the manuscript. After using this tool, the authors carefully reviewed and edited all content and take full responsibility for the final version of the publication.
