# Supplementary material for: Formative research to co-develop a Yoga-based intervention for antenatal depression in rural women in Maharashtra, India
Source: Wellcome Open Res. 2026 Jun 3;11:25. Originally published 2026 Jan 9. [Version 2] doi: 10.12688/wellcomeopenres.25422.2 (PMC13273246; doi:10.12688/wellcomeopenres.25422.2)
Supplement: Peer review history

# Reviewer report: Reviewer response for version 2

**DOI:** 10.21956/wellcomeopenres.29498.r157604

**Published:** 2026-06-16

**Authors:** Carolina Estevao

**Affiliations:** 1 Kings College London, London, UK

Elegantly described process of development of a prenatal yoga intervention in India.

 Great responsiveness to the initial review's comments.

 Outstanding use of HCD to guide this process, which is frequently absent in co-design methodology. The triangulation of data makes me confident that the findings are relevant and of the highest quality for the subsequent research participants.

 The manuscript lacks details regarding how these user-friendly resources will be distributed to low-literacy populations within rural settings.

 This paper is highly valuable to global mental health and implementation science literature and should be indexed.

Is the work clearly and accurately presented and does it cite the current literature?

Yes

If applicable, is the statistical analysis and its interpretation appropriate?

Not applicable

Are all the source data underlying the results available to ensure full reproducibility?

No source data required

Is the study design appropriate and is the work technically sound?

Yes

Are the conclusions drawn adequately supported by the results?

Yes

Are sufficient details of methods and analysis provided to allow replication by others?

Yes

Reviewer Expertise:

Yoga for mental health

I confirm that I have read this submission and believe that I have an appropriate level of expertise to confirm that it is of an acceptable scientific standard.

# Reviewer report: Reviewer response for version 1

**DOI:** 10.21956/wellcomeopenres.28008.r144667

**Published:** 2026-02-20

**Authors:** Tina Cartwright

**Affiliations:** 1 University of Westminster, London, UK

**Summary**

 This manuscript presents a well-conducted formative study to co-develop an antenatal Yoga intervention for antenatal depression in rural Maharashtra, India. The multi-phase design guided by a Human-Centred Design (HCD) framework is substantive, incorporating qualitative interviews, participatory development and intervention adaptation workshops, an intervention cohort (testing of intervention), community engagement activities, and a Theory of Change (ToC) workshop. The body of work demonstrates extensive and meaningful engagement with pregnant women, family members, community health workers, yoga instructors and other stakeholders, including the broader community – this is to be commended. The iterative refinement and testing of the yoga intervention and supporting materials is a further strength (though more detail is needed), and the research makes a valuable contribution to culturally grounded intervention development in maternal mental health. However, there are several areas that could be clarified/further developed to further strengthen the manuscript. Recommendations include enhancing clarity of presentation, strengthening engagement with the broader evidence base, and further clarifying the mechanisms/conceptual framing (ToC) and future trajectory of the research.

**Specific comments**

**Abstract/introduction**

 Framing of study and intervention – the title and aims indicates that the study aims “to codevelop a Yoga-based intervention for antenatal depression in rural women… However, much of the formative work suggests that it is geared towards prevention i.e. supporting rural women’s physical and mental health thereby reducing healthcare needs (as reported by some stakeholders/community workers) and risk of depression. Either way, it could be clearer throughout, including discussion around its future implementation (“for pregnant women”).

 Engagement with the wider literature – the manuscript would benefit from greater engagement with research exploring mechanisms of action for yoga-based interventions for maternal health and for reducing depressive symptoms, particularly since this relates to aim 3. This should be included in the introduction and ideally incorporated into the ToC, as discussed below.

 Please provide brief information about the Integrated Approach to Yoga Therapy during Pregnancy intervention as this provides the context for the current Yoga-Sanskar intervention development.

**Methods**

 Design - *“*The data from these phases were collated, triangulated, and synthesised, leading to the development of a range of intervention delivery materials and research outputs.” Please provide further details of how this was done i.e. what was the synthesis process, how were different types of data triangulated? It might be helpful to frame this within a mixed-methods (or multi-method) paradigm.

 It would be helpful to explanation of some context specific factors e.g. what are Anganwadi centres.

 As there are many different components of the formative research, it can sometimes be challenging as a reader to follow (when moving between methods and results), especially with multiple acronyms. I suggest that you include participant characteristics in the methods section for each component (rather than combined across all elements at start of the results section) - this indicates from outset, how many workshops etc were conducted and with whom which would aid clarity as well as showcasing each stage of the research. Or you could include in your visual representation.

 ToC workshop – can more detail of methodology be provided in supporting materials as with other stages of the development work.

 The ‘Intervention Cohorts’ evaluated the feasibility and practical implementation of the refined intervention – this is an important and valuable part of the research with 88 participants, but very limited detail is provided, either in the methods or results (were the 9 interviews analysed together with the data from the adaptation workshops?). Please provide more detail of the intervention duration, session frequency/duration, adherence etc. If the feasibility study is reported elsewhere in more detail (or there are plans to do so), then please state here.

 In the analysis section, please provide further details of thematic analysis process and provide relevant references. In the discussion, there is reference to reflexive engagement, rigour and positionality, but this needs to be clearly evidenced and explained within the methods.

**Results**

 Please provide the final Yoga-Sanskar intervention protocol (in table format, similar to the Satyapriya et al. study) – this could either be in the main manuscript or in a supplementary file. Can access to the intervention materials also be included and in the supporting materials? Also briefly clarify what adaptations were made to the original intervention content based on your formative research.

 The function of the measures developed from development workshops (CSIP and ATYS) was not clear – were these intended as exploratory process measures? Were pre-existing measures not suitable?

 At present, the ToC model focuses on operational steps and assumptions, it does not incorporate mechanisms. Consider strengthening the model by explicitly articulating hypothesised psychological and social mechanisms linking yoga participation to outcomes, and by distinguishing short, medium, and long-term outcomes in the model.

 Why 12 weeks – was this the length of the programme tested in the Intervention Cohort study or based on the previous intervention or broader research evidence?

**Discussion**

 The strengths of the research are clearly stated in relation to the focus on user needs and understanding contextual and cultural factors for successful intervention design and implementation. However, I suggest caution in overstating the methodological novelty of the study (paragraph 7):

 “This is probably the first study to have used the principles of co-development and HCD in the field of Yoga research. To the best of our knowledge, ToC approach is also being used for the first time in Yoga research…”

 Co-development and tailoring of yoga interventions with users and stakeholders is increasingly common practice – if using different frameworks (e.g. Medical Research Council complex interventions (MRC) framework). However, the authors are justified in highlighting the strengths of their particular approach to ensuring iterative intervention development with “the user at the heart of the process”. 

 As mentioned in methods comments, further information is needed to evidence some of the points raised in the discussion around methodological rigour and trustworthiness.

 I would like to see further elaboration of the implications for practice, including future implementation of the intervention – the ToC map suggests an RCT? 

 Minor comments

 Abstract - “Yoga is increasingly promoted within India’s public health system as an evidence-based mind–body intervention. *However, adoption of regular practice of Yoga is possible if we design culturally relevant and contextually appropriate implementation plan.” *Rephrase for clarity – I think you mean that important to tailor interventions in order to encourage regular practice and intervention effectiveness.

 Reduce the use of multiple acronyms to enhance readability.

Is the work clearly and accurately presented and does it cite the current literature?

Partly

If applicable, is the statistical analysis and its interpretation appropriate?

Not applicable

Are all the source data underlying the results available to ensure full reproducibility?

Partly

Is the study design appropriate and is the work technically sound?

Yes

Are the conclusions drawn adequately supported by the results?

Partly

Are sufficient details of methods and analysis provided to allow replication by others?

Partly

Reviewer Expertise:

Health psychology, yoga research, wellbeing, long-term conditions

I confirm that I have read this submission and believe that I have an appropriate level of expertise to confirm that it is of an acceptable scientific standard, however I have significant reservations, as outlined above.

# Response

**Published:** 2026-05-26

**Authors:** Rahul Shidhaye

**Affiliations:** Pravara Institute of Medical Sciences, India

**26 May 2026**

**Dear Dr Cartwright,**

**Manuscript Title:** **Formative research to co-develop a Yoga-based intervention for antenatal depression in rural women in Maharashtra, India.**

 Many thanks for taking the time to review our submission and providing very valuable comments and suggestions. We would like to thank the reviewers for very constructive feedback on the manuscript.  We have responded to your comments in italics below and believe we have been able to address all the comments. We have made corresponding changes to the manuscript and the revised manuscript is uploaded.

**Reviewer 1** Comment # 1 (Summary):  This manuscript presents a well-conducted formative study to co-develop an antenatal Yoga intervention for antenatal depression in rural Maharashtra, India. The multi-phase design guided by a Human-Centred Design (HCD) framework is substantive, incorporating qualitative interviews, participatory development and intervention adaptation workshops, an intervention cohort (testing of intervention), community engagement activities, and a Theory of Change (ToC) workshop. The body of work demonstrates extensive and meaningful engagement with pregnant women, family members, community health workers, yoga instructors and other stakeholders, including the broader community – this is to be commended. The iterative refinement and testing of the yoga intervention and supporting materials is a further strength (though more detail is needed), and the research makes a valuable contribution to culturally grounded intervention development in maternal mental health. However, there are several areas that could be clarified/further developed to further strengthen the manuscript. Recommendations include enhancing clarity of presentation, strengthening engagement with the broader evidence base, and further clarifying the mechanisms/conceptual framing (ToC) and future trajectory of the research.

**Response**: *We thank the reviewer for their constructive feedback on our manuscript. In response, we have made several revisions to improve the clarity and flow of the manuscript, strengthen the conceptual grounding of our program theory, situate our findings within the broader evidence base, and clarify the future directions of this work.*

Comment # 2:

 Framing of study and intervention – the title and aims indicates that the study aims “to codevelop a Yoga-based intervention for antenatal depression in rural women… However, much of the formative work suggests that it is geared towards prevention i.e. supporting rural women’s physical and mental health thereby reducing healthcare needs (as reported by some stakeholders/community workers) and risk of depression. Either way, it could be clearer throughout, including discussion around its future implementation (“for pregnant women”). **Response**: *We thank the reviewer for this important observation regarding the framing of the intervention. We agree that, while the primary aim of the YOGA-D program (described below) is to develop and evaluate a yoga-based intervention for antenatal depression, findings from the formative research also highlight its broader potential in promoting overall physical and mental well-being among pregnant women, thereby contributing to both prevention and management of depressive symptoms.* *In response, we have revised the third and fourth paragraphs of the Introduction to more clearly situate the formative research within the larger YOGA-D program and its implementation pathway. We have added the following text,* *‘* *In this context, the YOGA-D (Yoga-based Lifestyle Intervention for Antenatal Depression) program was initiated to explore the potential of a Yoga-based intervention to improve maternal mental health among pregnant women in rural Maharashtra *(Shidhaye et al., 2024) *. YOGA-D is a five-year, multi-phase research program comprising an initial formative research phase to co-develop the intervention, followed by a pilot randomized controlled trial to assess its feasibility and acceptability, and a subsequent pragmatic randomized controlled trial to evaluate its effectiveness and cost-effectiveness.* *This paper presents the rationale, process, and key findings from the formative research phase. The primary output of this phase is the Yoga-Sanskar (YS) intervention (described below), which will be further evaluated for its feasibility, acceptability, and effectiveness. The protocol for the pilot randomized controlled trial has been published previously *(Shidhaye et al., 2024) *, and clinical trial registration details for the pragmatic randomized controlled trial are available online *(Clinical Trials Registry of India, 2024 *)* *.* *In addition, we have clarified this positioning in the Discussion section by explicitly linking the formative research to the planned evaluation phases. The modified text is as follows,* *‘* *The formative research phase led to the co-development of the Yoga-Sanskar intervention. We plan to evaluate the feasibility, acceptability, and effectiveness of the intervention using a pilot randomized controlled trial *(Shidhaye et al., 2024) *and the pragmatic randomized controlled trial *(Clinical Trials Registry of India, 2024) *.’*

Comment # 3:

 Engagement with the wider literature – the manuscript would benefit from greater engagement with research exploring mechanisms of action for yoga-based interventions for maternal health and for reducing depressive symptoms, particularly since this relates to aim 3. This should be included in the introduction and ideally incorporated into the ToC, as discussed below.

**Response**: *We thank the reviewer for this important suggestion and agree that engaging more explicitly with the literature on the mechanisms of action of yoga-based interventions strengthens the study's conceptual grounding. In response to this comment and a related comment below (comment #15), we have incorporated a brief discussion of potential biological and psychosocial mechanisms into the manuscript.* *Specifically, we have added the following text to the Methods section,* *‘ToC was primarily used to describe the programmatic aspects and contextual factors, and not to explain the biological mechanism of action. It is likely that regular Yoga practice may reduce stress through the modulation of the hypothalamic-pituitary-adrenal axis. Psychosocial mechanisms such as improved mindfulness and self-awareness (mind–body awareness), enhanced emotional regulation (self-regulation), and increased physical self-efficacy and fitness may contribute to improvements in mood and quality of life, as described by Butzer et al.*(Butzer et al., 2016) *. These mechanisms are described in detail in our previously published protocol *(Shidhaye et al., 2024) *and will be assessed as part of the randomized controlled trial.’* *In the current study, the ToC was primarily used to map the operational steps linking interventions to outcomes, while accounting for contextual factors and assumptions influencing implementation. As the ToC is an output of the co-development process, its primary focus is on programmatic pathways. While primarily focused on programmatic pathways, the model is indeed informed by hypothesised psychosocial mechanisms, including self-awareness, self-efficacy, and social support, which are intended to be assessed in the pragmatic randomized controlled trial. Please also see our response to comment#15 below.*

Comment # 4:

 Please provide brief information about the Integrated Approach to Yoga Therapy during Pregnancy intervention as this provides the context for the current Yoga-Sanskar intervention development.

**Response**: *We thank the reviewer for this suggestion. We have included the following in the Introduction section,* *‘* *We used the* ***Integrated Approach to Yoga Therapy (IAYT) during Pregnancy*** (Satyapriya et al., 2013) *, as an initial evidence-based intervention and collaboratively adapted its content to co-develop the* ***Yoga-Sanskar (YS)*** *intervention. IAYT module for pregnant women was developed based on classical texts, including the Patanjali Yoga Sutras and the Mandukya Karika. It adopts a holistic approach to health, addressing physical, mental, emotional, and intellectual well-being, and uses specific techniques to enhance mental equilibrium. Participants were trained in the complete yoga sequence by certified instructors through structured sessions of two hours per day, three days per week, over one month. Refresher sessions were conducted during routine antenatal visits, and participants were encouraged to continue regular home practice using recorded materials. The IAYT intervention demonstrated significant reductions in anxiety and depressive symptoms among pregnant women *(Satyapriya et al., 2013) *. IAYT intervention was implemented and evaluated in an urban, hospital-based setting with intensive initial training and facility-based Yoga sessions.’*  

Comment # 5:

 “The data from these phases were collated, triangulated, and synthesised, leading to the development of a range of intervention delivery materials and research outputs.” Please provide further details of how this was done i.e. what was the synthesis process, how were different types of data triangulated? It might be helpful to frame this within a mixed-methods (or multi-method) paradigm.

**Response**: *We have added the following details in section 2.9 (Synthesis of findings and Development of intervention materials),* *“* *We first documented the findings from co-development workshops, intervention cohorts, and community engagement activities using detailed field notes, participant outputs (e.g., participatory exercises), and facilitator reflections. The next step was synthesis of findings, and this was done using iterative team discussions. Findings from each data source were compared and contrasted to identify areas of convergence, complementarity, and divergence. For example, themes related to barriers, facilitators, and perceptions of Yoga identified in qualitative interviews were cross-checked with insights emerging from co-development workshops and intervention cohorts. This process helped us to understand context-specific nuances that informed intervention design. Triangulated findings were then mapped onto the outcomes’ pathway within the Theory of Change framework. These insights directly informed the development of intervention delivery strategies, session content, and supporting materials (e.g., Yoga-Sanskar chart, Yoga-Dainandini, and digital resources). This integrative approach ensured that the final intervention was grounded in both empirical evidence and participant-driven insights. The details are in the results section below.”* *The Results subsection, “Synthesis of Findings and Development of Intervention Materials,” provides additional detail on how insights from the various formative research activities were integrated into the design of the intervention materials.*

Comment # 6:

 It would be helpful to explanation of some context specific factors e.g. what are Anganwadi centres.  

**Response**: *We thank the reviewer for this suggestion. We have included the following in the Methods section,* *‘Anganwadi centres are community-based centres established under the Integrated Child Development Services (ICDS) programme that provide a range of essential health and nutrition services, including basic health check-ups, referrals, supplementary nutrition, immunisation, and non-formal pre-school education. Their primary beneficiaries include pregnant and lactating women, children, and adolescent girls.’*

Comment # 7:

 As there are many different components of the formative research, it can sometimes be challenging as a reader to follow (when moving between methods and results), especially with multiple acronyms. I suggest that you include participant characteristics in the methods section for each component (rather than combined across all elements at start of the results section) - this indicates from outset, how many workshops etc were conducted and with whom which would aid clarity as well as showcasing each stage of the research. Or you could include in your visual representation. ToC workshop – can more detail of methodology be provided in supporting materials as with other stages of the development work.  

**Response**: *We have now included participant characteristics in the Methods sections, and the ToC workshop details are added as follows,* *‘The workshop was conducted in person at the Clinical Trial Centre of Pravara Institute of Medical Sciences and lasted approximately 2-3 hours. Discussions were conducted in the local language (Marathi) to facilitate active participation from all the stakeholders. Field notes were taken by trained researchers, and the ToC diagrams developed by each group were documented for analysis.* *The session commenced with a brief orientation on the ToC framework by the PI who facilitated the workshop. A hypothetical example was presented to illustrate the process of back-mapping from a defined long-term outcome to its necessary preconditions. This was followed by a detailed explanation of how ToC facilitates causal reasoning in complex community-based interventions.* *Subsequently, participants were divided into three groups, each guided by trained co-facilitators with prior orientation to ToC methodology. All groups were assigned a common outcome statement: “Pregnant women practicing Yoga regularly for three months.” Each group constructed a preliminary ToC diagram that included the following components: (i) activities and outputs required to initiate change, (ii) short- and intermediate-term outcomes, (iii) long-term intended outcomes, and (iv) underlying assumptions and barriers that could influence the causal pathway.* *Each group presented its ToC map to the larger group in a plenary discussion. Feedback was solicited to identify points of convergence and contextual variation across groups. The three ToC maps were then synthesised by RS (first author) into a single consolidated version, which the co-authors subsequently reviewed for internal consistency and conceptual coherence.’*

Comment # 8:  The ‘Intervention Cohorts’ evaluated the feasibility and practical implementation of the refined intervention – this is an important and valuable part of the research with 88 participants, but very limited detail is provided, either in the methods or results (were the 9 interviews analysed together with the data from the adaptation workshops?). Please provide more detail of the intervention duration, session frequency/duration, adherence etc. If the feasibility study is reported elsewhere in more detail (or there are plans to do so), then please state here.

**Response**: *We thank the reviewer for this important suggestion and have added the following in Section 2.6 (Intervention Cohort),* *‘A total of 72 participants were invited to join the intervention cohort, of whom 45 agreed to participate (62.5%). A trained yoga instructor conducted group yoga sessions in Anganwadi centres. Each session lasted approximately 60–70 minutes, with five consecutive daily sessions in the first week followed by weekly sessions thereafter. About half of the participants (23) attended between three to five sessions, while the remaining participants attended only one to two sessions. Additionally, 20 participants attended between one to six weekly follow-up sessions. Attendance was monitored through session records maintained by the study team. To assess the acceptability and participant experience of the intervention, in-depth interviews were conducted with nine participants following completion of the follow-up yoga sessions. These interviews were analyzed separately from the adaptation workshop data. A more detailed feasibility assessment, including process evaluation, is being conducted as part of a pilot randomized controlled trial, for which the protocol has been published *(Shidhaye et al., 2024) *and the findings the pilot randomized controlled trial are currently under review *(Clinical Trials Registry of India, 2024) *.*

Comment # 9:

 In the analysis section, please provide further details of thematic analysis process and provide relevant references.  

**Response**: *We apologise for not including adequate details in the initial submission. This was primarily due to concerns about the word limit. We have now included the following details in the manuscript under section 2.11 (Data management and analysis).* *“We employed thematic content analysis to examine and synthesize the data *(Anderson, 1997; Braun & Clarke, 2012) *. Members of the research team (RS, ST, SP, KS, and PN) repeatedly reviewed the transcripts in both English and Marathi to achieve familiarity with the data and met regularly to discuss emerging insights. An initial coding framework was developed after SP and ST independently coded two transcripts. This draft codebook was subsequently reviewed and refined in consultation with RS.* *Coding responsibilities were then divided: SP independently coded approximately half of the transcripts, which were later reviewed by ST, while ST coded the remaining transcripts, followed by review from SP. Additional codes were generated inductively as new patterns emerged from the data. Any discrepancies in coding were discussed and resolved collaboratively among RS, SP, and ST. The broader research team (RS, KS, SP, ST, PN, and SG) participated in reviewing the coded data and identifying key themes. NVivo 9 (QSR International) was used to organize, manage, and analyze the data throughout the process.”*  

Comment # 10:

 In the discussion, there is reference to reflexive engagement, rigour and positionality, but this needs to be clearly evidenced and explained within the methods.

**Response**: *We again apologise for omitting these important details from the methodology section. We have now added the following section under the heading, ‘Reflexivity and positionality’ in the methods section. We thank the reviewer for this important comment.* *“The data were collected, transcribed, translated, coded, and analyzed by a team of researchers familiar with the local cultural context. CT conducted all IDIs and FGDs. She holds a master’s degree in medical social work and clinical psychology and is currently pursuing a doctoral degree. At the time of data collection, she also served as the intervention coordinator, overseeing the intervention adaptation process. CT is a native Marathi speaker and shares the sociocultural background of the participants, which facilitated rapport-building and contextual understanding. She had prior experience in conducting structured interviews and received additional training from RS in qualitative data collection methods.* *Other members of the research team (KS, SP, ST, PN, and SG) are also native Marathi speakers with a strong understanding of the local context and were involved in various stages of qualitative data analysis. RS has previous experience in leading qualitative research studies.* *At the beginning of each interaction, CT introduced herself and explained the study objectives to participants. While her dual role as a researcher and intervention coordinator may have influenced participants’ responses and data interpretation, efforts were made throughout the research process to maintain reflexivity through team-based discussions, iterative analysis, and critical review of emerging findings.”*  

Comment # 11:

 Please provide the final Yoga-Sanskar intervention protocol (in table format, similar to the Satyapriya et al. study) – this could either be in the main manuscript or in a supplementary file.  

**Response**: *We apologise for not including this in the earlier submission. We have now included the Yoga-Sanskar protocol in a table format as supplementary file 2.*

Comment # 12:

 Can access to the intervention materials also be included and in the supporting materials? **Response**: *We have now uploaded the intervention materials as a supplementary file on figshare. The link for the file: https://doi.org/10.6084/m9.figshare.30665477.v2*  

Comment # 13:

 Also briefly clarify what adaptations were made to the original intervention content based on your formative research.  

**Response**: *This is an important suggestion and we thank the reviewer for the same. In response, we have added section 3.7 at the end of the Results section. The text is as below,* ***‘3.7 Summary of adaptations made to the original intervention*** *Based on feedback from both experts and participants during the formative research phase, several adaptations were made to the original Integrated Approach of Yoga Therapy during Pregnancy (IAYT-P) to enhance feasibility, acceptability, and adherence in the local context. The original yoga module was simplified to improve safety and suitability for pregnant women in community settings. Specific practices were removed, including Gulpha vistara svasanam (ankle stretch breathing), Trikonasana (triangle pose), Ardha pavanamuktasana (folded leg lumbar stretch), and pranayama techniques such as Sheetali. In contrast, gentle and supportive practices such as Griva Sanchalan (neck movements) and Supta-baddha Konasana were included to enhance comfort and appropriateness during pregnancy.* *Findings from the intervention adaptation workshops further highlighted the need to incorporate behaviour change strategies alongside the yoga content, leading to modifications in the structure and delivery of the intervention. The programme was reorganised into two phases: a LEARN phase and a PRACTICE phase, to support gradual skill acquisition and sustained engagement. The LEARN phase consisted of directly supervised group sessions led by trained yoga instructors, beginning with an initial orientation session focused on building commitment, explaining the benefits and safety considerations of yoga during pregnancy, and demonstrating the full yoga sequence. This was followed by three additional supervised sessions conducted on consecutive days, during which participants were taught individual components of the sequence, including sukshma-vyayam, selected asanas, pranayama, and relaxation techniques, with emphasis on correct technique, breath awareness, and safety. Participants also received individualised feedback and modifications based on their comfort and ability.* *The PRACTICE phase began alongside the LEARN phase and extended over three months, during which participants were encouraged to progressively transition to independent home-based practice. A competency assessment was conducted within three to four weeks after the first session to ensure safe and correct practice. Weekly group sessions were conducted during this phase to reinforce learning, address challenges, and maintain motivation. The duration and structure of sessions were standardised, with supervised sessions lasting approximately 60–75 minutes. To further address barriers to adherence identified during formative research, several supportive components were integrated as described above. These included a yoga diary (Yoga Dainandini) with structured home practice guidance and self-monitoring, QR code–linked video demonstrations, a yoga manual, and educational leaflets on physical activity, sleep, and diet during pregnancy. In addition, a trained community-based facilitator (Sahayogini) provided ongoing motivation and support through regular interactions. Together, these adaptations ensured that the intervention was contextually appropriate, participant-centred, and feasible for implementation in real-world settings.’*

Comment # 14:

 The function of the measures developed from development workshops (CSIP and ATYS) was not clear – were these intended as exploratory process measures? Were pre-existing measures not suitable?  

**Response**: *We thank the reviewer for this important question and for seeking clarification on the role of the measures developed during the formative phase.* *The Common Symptoms in Pregnancy (C-SIP) inventory and the Attitude towards Yoga Practice during Pregnancy (ATYP) scale were developed as **context-specific, exploratory measures** to inform both intervention design and implementation, rather than as validated psychometric tools for measuring primary outcomes.* *During the in-depth interviews and intervention development workshops, pregnant women reported a range of physical, psychological, and interpersonal concerns. Many of these were common, non-life-threatening symptoms that nonetheless had a significant impact on their daily lives. The 51-item C-SIP inventory was developed to systematically capture these “felt needs” of participants and to ensure that the yoga intervention remained responsive and relevant to their lived experiences. In addition, it was used to facilitate rapport-building and engagement, with yoga instructors encouraged to discuss individual concerns and link them to the practice of yoga. However, during the pilot randomized trial, administration of the full C-SIP tool proved operationally challenging. As a result, key elements of symptom monitoring were subsequently integrated into the Yoga-Dainandini to support ongoing self-reflection and tracking in a more feasible manner.* *Similarly, the ATYP scale was developed to assess participants’ attitudes towards yoga practice during pregnancy, which we identified as a key determinant of intervention uptake and adherence. The scale captures domains such as awareness of prenatal yoga, perceived benefits and harms, emotional responses to yoga, and anticipated barriers to regular practice. We were unable to identify an existing validated measure that adequately captured these context-specific constructs in pregnant women in rural India, particularly in relation to yoga practice. Therefore, the ATYP scale was developed to serve as an exploratory process measure to better understand factors influencing engagement with the intervention.*

Comment # 15:

 At present, the ToC model focuses on operational steps and assumptions, it does not incorporate mechanisms. Consider strengthening the model by explicitly articulating hypothesised psychological and social mechanisms linking yoga participation to outcomes, and by distinguishing short, medium, and long-term outcomes in the model.  

**Response**: *We thank the reviewer for this insightful comment and agree that explicitly articulating mechanisms and outcome pathways can strengthen the Theory of Change (ToC).* *In the current study, the ToC primarily focuses on mapping the operational steps, intermediate and long-term outcomes, the interventions linking these outcomes, and the contextual factors and assumptions that may influence their achievement. This approach is consistent with the methodology outlined by the Center for Theory of Change, which defines ToC as “a comprehensive description and illustration of how and why a desired change is expected to happen in a particular context,” with a particular emphasis on mapping the “missing middle” between program activities and long-term outcomes *(Breuer et al., 2016; *Centre for Theory of Change*, n.d.) *In our study, we conceptualized the ToC as a programme theory/small theory to explain the implementation pathway-how the intervention/project/policy is expected to work in practice *(Breuer et al., 2016) *rather than to provide a detailed account of the underlying biological or psychological mechanisms of action of Yoga on depression outcomes. However, we acknowledge that articulating these mechanisms can enhance the explanatory value of the model.* *There is emerging evidence to suggest that Yoga may influence mental health through multiple pathways. For example, regular Yoga practice may reduce stress through modulation of the hypothalamic-pituitary-adrenal axis. However, we are not testing this in our study.* *Additionally, psychosocial mechanisms such as improved mindfulness and self-awareness (mind–body awareness), enhanced emotional regulation (self-regulation), and increased physical self-efficacy and fitness may contribute to improvements in mood and quality of life, as described by Butzer et al. *(Butzer et al., 2016) *. These mechanisms were described in detail in our previously published protocol *(Shidhaye et al., 2024) *and we have included ‘self-awareness’ as one of the intermediate outcomes linking Yoga practice with reduction in depression.* *In response to the reviewer’s suggestion, we have now clarified in the Methods section that the ToC primarily addresses programmatic and contextual pathways and not the biological mechanism of action. The following text is included,* *‘ToC was primarily used to describe the programmatic aspects and contextual factors, and not to explain the biological mechanism of action. It is likely that regular Yoga practice may reduce stress through the modulation of the hypothalamic-pituitary-adrenal axis. Psychosocial mechanisms such as improved mindfulness and self-awareness (mind–body awareness), enhanced emotional regulation (self-regulation), and increased physical self-efficacy and fitness may contribute to improvements in mood and quality of life, as described by Butzer et al. *(Butzer et al., 2016) *. These mechanisms are described in detail in our previously published protocol *(Shidhaye et al., 2024) *, and will be assessed as part of the randomized controlled trial’ *  

Comment # 16:

 Why 12 weeks – was this the length of the programme tested in the Intervention Cohort study or based on the previous intervention or broader research evidence?  

**Response**: *We thank the reviewer for this important question regarding the rationale for the 12-week duration of the intervention.* *The duration was informed by both methodological and contextual considerations. In clinical trials for depression, primary outcomes are commonly assessed at approximately 12 weeks (three months) post-randomization, as this timeframe is considered sufficient to observe clinically meaningful changes in depressive symptoms *(Cuijpers et al., 2014; *National Institute for Health and Care Excellence (NICE)*, 2022) *.This is also consistent with broader evidence on yoga interventions during pregnancy; for instance, the systematic review by *(Corrigan et al., 2022) *suggests that interventions are often delivered over similar durations.* *In addition, the 12-week period aligns well with the antenatal care context in rural India. Pregnancy is typically registered between 10–12 weeks of gestation, making this a practical entry point for initiating the intervention. A 12-week duration corresponds broadly to the second trimester, which is generally considered a safe and appropriate period for engaging in structured physical activity, including yoga. Furthermore, in many rural settings, women often relocate to their maternal homes around the seventh month of pregnancy for delivery, which may limit continuity of participation beyond this period.* *Taken together, the 12-week duration was selected to balance clinical relevance, behavioral change, and feasibility within the local sociocultural and healthcare context.*

Comment # 17:

 The strengths of the research are clearly stated in relation to the focus on user needs and understanding contextual and cultural factors for successful intervention design and implementation. However, I suggest caution in overstating the methodological novelty of the study (paragraph 7): “This is probably the first study to have used the principles of co-development and HCD in the field of Yoga research. To the best of our knowledge, ToC approach is also being used for the first time in Yoga research…” Co-development and tailoring of yoga interventions with users and stakeholders is increasingly common practice – if using different frameworks (e.g. Medical Research Council complex interventions (MRC) framework). However, the authors are justified in highlighting the strengths of their particular approach to ensuring iterative intervention development with “the user at the heart of the process”.

**Response**: *We agree with the reviewer and apologise for overstating the methodological novelty of this study. We have now deleted that sentence and replaced it with the following in the discussion section of the manuscript,* *‘While co-development, Human-Centered Design (HCD), and Theory of Change (ToC) approaches are increasingly used in public health research, their application within yoga-based interventions remains relatively underexplored. This study is one of the very few to apply these approaches in this context.’*  

Comment # 18:

 As mentioned in methods comments, further information is needed to evidence some of the points raised in the discussion around methodological rigour and trustworthiness.  

**Response**: *We thank the reviewer for this important comment. In response, we have made several revisions throughout the manuscript to enhance methodological clarity and strengthen the trustworthiness and transparency of our findings. Key changes include the addition of a clearer description of the overarching goals of the YOGA-D program in the Introduction to better frame the study objectives. We have also included a detailed description of the Integrated Approach to Yoga Therapy (IAYT) that informed the co-development of the Yoga-Sanskar intervention, and further elaborated on the purpose and conduct of the Theory of Change workshops.* *In the Methods section, we have clarified the use of data triangulation across multiple formative research activities, investigator triangulation through team-based coding and review, and iterative discussions used to refine codes and themes. We have also expanded the description of the coding process, including the development and refinement of the codebook and the resolution of discrepancies through consensus. In addition, the reflexivity section has been strengthened to better acknowledge researcher positionality and its potential influence on data collection and interpretation.* *Finally, in the Discussion, we have clarified the progression of the study beyond the co-development phase and moderated claims regarding methodological novelty. We believe these revisions improve transparency and provide a clearer account of the analytical process, thereby strengthening the overall trustworthiness of the study findings.*  

Comment # 19:

   I would like to see further elaboration of the implications for practice, including future implementation of the intervention – the ToC map suggests an RCT?

**Response**: *We sincerely apologise for not including these details in the earlier version of the manuscript. We have now added the following statement to the first paragraph of the Discussion section,* *‘The formative research phase led to the co-development of the Yoga-Sanskar intervention. We plan to evaluate the feasibility, acceptability, and effectiveness of the intervention using a pilot randomized controlled trial *(Shidhaye et al., 2024) *and the pragmatic randomized controlled trial design *(Clinical Trials Registry of India, 2024) *. *  

Comment # 20:  Abstract - “Yoga is increasingly promoted within India’s public health system as an evidence-based mind–body intervention. However, adoption of regular practice of Yoga is possible if we design culturally relevant and contextually appropriate implementation plan.” Rephrase for clarity – I think you mean that important to tailor interventions in order to encourage regular practice and intervention effectiveness.

**Response**: *We thank the reviewer for this suggestion and have made the following changes in the abstract,* *‘Yoga is increasingly promoted within India’s public health system as an evidence-based mind–body intervention.* *However, its sustained adoption and effectiveness depend on designing culturally relevant and contextually appropriate interventions.’*  

Comment # 21:

 Reduce the use of multiple acronyms to enhance readability. 

**Response**: *We have made appropriate changes in the revised manuscript.*

   Anderson, R. (1997). *Thematic Content Analysis ( TCA ) 1 Descriptive Presentation of Qualitative Data*. 1–4. Braun, V., & Clarke, V. (2012). Thematic analysis. In *APA handbook of research methods in psychology, Vol 2: Research designs: Quantitative, qualitative, neuropsychological, and biological.* (pp. 57–71). American Psychological Association. https://doi.org/10.1037/13620-004 Breuer, E., De Silva, M. J., Shidaye, R., Petersen, I., Nakku, J., Jordans, M. J. D., Fekadu, A., & Lund, C. (2016). Planning and evaluating mental health services in low-and middle-income countries using theory of change. *British Journal of Psychiatry*. https://doi.org/10.1192/bjp.bp.114.153841 Butzer, B., Bury, D., Telles, S., & Khalsa, S. B. S. (2016). Implementing yoga within the school curriculum: A scientific rationale for improving social-emotional learning and positive student outcomes. *Journal of Children’s Services*, *11*(1), 3–24. https://doi.org/10.1108/JCS-10-2014-0044 *Centre for Theory of Change*. (n.d.). Clinical Trials Registry of India. (2024). *A Pragmatic Randomized Controlled Trial to assess the effectiveness and cost-effectiveness of Yoga for Antenatal Depression in Maharashtra, India (PRAGYA).* Corrigan, L., Moran, P., McGrath, N., Eustace-Cook, J., & Daly, D. (2022). The characteristics and effectiveness of pregnancy yoga interventions: a  systematic review and meta-analysis. *BMC Pregnancy and Childbirth*, *22*(1), 250. https://doi.org/10.1186/s12884-022-04474-9 Cuijpers, P., Karyotaki, E., Weitz, E., Andersson, G., Hollon, S. D., & van Straten, A. (2014). The effects of psychotherapies for major depression in adults on remission,  recovery and improvement: a meta-analysis. *Journal of Affective Disorders*, *159*, 118–126. https://doi.org/10.1016/j.jad.2014.02.026 *National Institute for Health and Care Excellence (NICE)*. (2022). Satyapriya, M., Nagarathna, R., Padmalatha, V., & Nagendra, H. R. (2013). Effect of integrated yoga on anxiety, depression & well being in normal pregnancy. *Complementary Therapies in Clinical Practice*, *19*(4), 230–236. https://doi.org/10.1016/J.CTCP.2013.06.003 Shidhaye, R., Bangal, V., Bhargav, H., Thanage, C., Gore, S., Talole, S., Shinde, K., Palande, S., Thete, U., Shelke, S., Gholap, G., Nisal, S., Gargade, S., Tilekar, S., Behere, N., Game, K., Murhar, V., Kunkulol, R., & Telles, S. (2024). Yoga-based lifestyle intervention for antenatal depression (YOGA-D): study  protocol for a pilot randomized controlled trial. *Wellcome Open Research*, *9*, 326. https://doi.org/10.12688/wellcomeopenres.22493.2

# Reviewer report: Reviewer response for version 1

**DOI:** 10.21956/wellcomeopenres.28008.r145238

**Published:** 2026-01-29

**Authors:** Prof Dr Meena Ramanathan

**Affiliations:** 1 Sri Balaji Vidyapeeth (Deemed to be University), Puducherry, India

**Study design is partly appropriate**

 Suggestion: Although the Yoga-Sanskar sequence and materials are described, replication would be enhanced by a concise tabular summary of the final Yoga protocol (postures, duration, frequency, trimester-specific adaptations)

**Replication:**

 Include brief clarification on facilitator training, session fidelity checks, and how consistency was maintained across multiple workshops/instructors.

**Summary of the article:**

 This article reports a multi-phased formative study that used human-centred design and Theory of Change approaches to co-develop a culturally appropriate Yoga-based intervention (Yoga-Sanskar) for antenatal depression among rural women in Maharashtra, India. Through qualitative interviews, participatory co-development and adaptation workshops, a feasibility cohort, community engagement activities, and stakeholder feedback, the authors systematically adapted an evidence-based prenatal Yoga protocol to local social, cultural, and practical contexts. The study demonstrates that the intervention is feasible, acceptable, and contextually relevant, highlights key barriers and facilitators to Yoga practice during pregnancy, and provides a transparent foundation for subsequent effectiveness testing and scale-up within public health settings.

Is the work clearly and accurately presented and does it cite the current literature?

Yes

If applicable, is the statistical analysis and its interpretation appropriate?

Yes

Are all the source data underlying the results available to ensure full reproducibility?

Yes

Is the study design appropriate and is the work technically sound?

Partly

Are the conclusions drawn adequately supported by the results?

Yes

Are sufficient details of methods and analysis provided to allow replication by others?

Partly

Reviewer Expertise:

Yoga, Yoga therapy, integrative medicine, Salutogenesis and complementary medicine, mental health, health and wellbeing

I confirm that I have read this submission and believe that I have an appropriate level of expertise to confirm that it is of an acceptable scientific standard.

# Response

**Published:** 2026-05-26

**Authors:** Rahul Shidhaye

**Affiliations:** Pravara Institute of Medical Sciences, India

26 May 2026

 Dear Prof Ramanathan, 

 Many thanks for your constructive feedback on our manuscript. We have now made several revisions to improve the clarity and flow of the manuscript, strengthen the conceptual grounding of our program theory, situate our findings within the broader evidence base, and clarify the future directions of this work.

 In response to your comment, we have now included the adapted yoga sequence (Yoga-Sanskar intervention) in supplementary file 2. We have also added further details on the activities undertaken during the workshops in the methods and results section.
